# Valorization of *Sargassum* Biomass as Potential Material for the Remediation of Heavy-Metals-Contaminated Waters

**DOI:** 10.3390/ijerph20032559

**Published:** 2023-01-31

**Authors:** Lázaro Adrián González Fernández, Amado Enrique Navarro Frómeta, Candy Carranza Álvarez, Rogelio Flores Ramírez, Paola Elizabeth Díaz Flores, Ventura Castillo Ramos, Manuel Sánchez Polo, Francisco Carrasco Marín, Nahum Andrés Medellín Castillo

**Affiliations:** 1 Multidisciplinary Postgraduate Program in Environmental Sciences, University Zone, Av. Manuel Nava 201, 2nd. Floor, San Luis Potosí 78000, Mexico; 2 Department of Inorganic Chemistry, Faculty of Science, University of Granada, 18071 Granada, Spain; 3 Food and Environmental Technology Department, Technological University of Izúcar de Matamoros, De Reforma 168, Campestre la Paz, Izúcar de Matamoros 74420, Mexico; 4 Faculty of Professional Studies Huasteca Zone, Universidad Autónoma de San Luis Potosí, Romualdo del Campo 501, Rafael Curiel, Ciudad Valles 79060, Mexico; 5 Coordination for the Innovation and Application of Science and Technology, Av. Sierra Leona # 550, Col. Lomas 2a. Sección, San Luis Potosí 78210, Mexico; 6 Faculty of Agronomy and Veterinary Medicine, Universidad Autónoma de San Luis Potosí, Carretera San Luis Potosí—Matehuala Km. 14.5 Ejido Palma de la Cruz, Soledad de Graciano Sánchez 78321, Mexico; 7 Center for Research and Postgraduate Studies, Faculty of Engineering, Universidad Autónoma de San Luis Potosí, Dr.Manuel Nava No. 8, West University Zone, San Luis Potosí 78290, Mexico

**Keywords:** *Sargassum* algae, valorization, adsorption, heavy metals, water remediation

## Abstract

*Sargassum* algae has become a major environmental issue due to its abundance in the Pacific Ocean with hundreds of tons reaching the beaches of the Mexican Caribbean every year. This generates large quantities of decomposing organic matter that have a negative impact on the region’s economy and ecosystems. *Sargassum* valorization has turned out to be a fundamental aspect to mitigate its environmental impact. This study proposes the use and application of untreated *Sargassum* biomass for the decontamination of waters polluted with lead (Pb) and cadmium (Cd) through single and binary adsorption tests. Physicochemical and textural properties examined by SEM, XRD, and FT-IR elucidated that *Sargassum* biomass is viable to be used as a potential environmental benign adsorbent, exhibiting Cd(II) and Pb(II) adsorption capacities as high as 240 mg g^−1^ and 350 mg g^−1^, respectively, outperforming conventionally used adsorbents. This is attributed to its morphology, favorable surface charge distribution, and the presence of -OH and -COH groups. A strong affinity between the biomass and metal pollutants was evidenced by a thermodynamics study, showing a spontaneous and endothermic process. This work sets a practical route for the utilization of the *Sargassum* biomass, demonstrating its applicability as a potential material for heavy-metal-polluted water remediation, making a substantial contribution to a circular economy system.

## 1. Introduction

The contamination of water resources is a widespread phenomenon and drinking water can often lose its original purpose. Direct and indirect sources of water pollution can be identified. The former includes effluents released by industries and refineries and the latter includes pollutants entering the water supply from the soil via groundwater systems or from the atmosphere via rainwater [1,2]. Contaminants of particular concern include metals derived from mining, metallurgy, electronics, electroplating, and metal finishing, among other industries [3]. The presence of heavy metal ions in industrial effluents is particularly disadvantageous because of their toxicity to organisms at multiple levels of the food chain [4].

To eliminate or reduce heavy metals to levels that do not represent a risk for human consumption, many adsorbents have been used. Table 1 shows some of these with their corresponding adsorption capacities.

Marine algae grow naturally on the continental shelves of seas and oceans. No benefit is derived from the marine algae that often cover Pacific coastal shores, where they are not only unsightly but generate offensive odors over time. *Sargassum*, a type of algae that regularly washes up on Caribbean beaches, has emerged as a major problem for water quality and has been described as an uncontrolled algae epidemic posing the greatest environmental challenge faced by Mexico. This genus of brown macroalgae (in the order Fucales) has impacted coastal ecosystems in the Mexican Caribbean, causing the death of turtles, fish, and other marine species [15]. It also represents a threat to human health from its decomposition on beaches and high content of arsenic and heavy metals, with negative economic effects on tourist activities in the region. *Sargassum* itself is not problematic but requires regulation and could even be used to benefit the environment, for example as a soil fertilizer [16].

Methodologies have recently been developed to adsorb pollutants such as heavy metals using materials of biological origin, including bacteria, algae, and fungi, or industrial, agricultural, and urban waste, potentially offering high viability and removal efficiency at a low cost. One of these techniques is biosorption, involving the selective transfer of one or more solutes from liquid phase to a batch of solid particles of biological material by various physical and chemical mechanisms [17]. Biosorption of heavy metals in algae and seagrasses is largely attributed to the properties of their cell walls, where a major role is played by electrostatic attraction and the formation of complex compounds. The cell walls of algae and seagrasses typically have a fibrillar skeleton, mostly composed of cellulose, and an amorphous matrix mainly formed by alginic acid or its salt (alginate) and a smaller number of sulfated polysaccharides (fucoidans) [18].

There is a wide diversity of marine algae, and the adsorption capacity and selectivity against various heavy metals have been found to differ among red, green, and brown algae. The adsorption of certain metals was shown to depend on the chemical composition of algae and the presence of different adsorption centers (fucoidans, alginates, phosphate proteins, etc.) and on their size, degree of solvation, and presence of chelating ions and molecular sieves, and on ion exchange with the species in the algae [18].

The main objective of this study was to characterize the biomass of the genus *Sargassum* and its usefulness to remove cadmium (Cd) and lead (Pb) from water by means of mono- and multi-component batch adsorption systems.

The results obtained could demonstrate that *Sargassum* biomass is a suitable option for the removal of metallic contaminants such as Pb and Cd and could replace other traditional adsorbent materials. The study of the composition and the physical and chemical properties of this biomass is necessary to determine if it is a highly promising adsorbent whose use could contribute to reduce not only the damage that it currently causes to the economy and coastal ecosystems of the Caribbean region of Mexico but also the problems of contamination by heavy metals.

## 2. Materials and Methods

### 2.1. Collection and Prior Treatment of Sargassum

*Sargassum* buxifolium was collected in situ from the algal biomass reaching the Mexican Caribbean based on the taxonomic and morphological characteristics described for the region in the specialist literature [19]. After their selection, samples were washed in abundant sea water and dried in the sun for 72 h. They were then packed in polyethylene bags for storage in the dark and under controlled humidity conditions.

Before their analysis, algae samples were thoroughly washed with distilled water to remove salts and other impregnated solid residues, dried in a natural ventilation oven at 60 °C for 24 h, and ground in an electric mill. They were then passed through vibrating nylon sieves of 30 to 50 µm, and fractions of each particle size were packed in high-density polyethylene bottles [20].

### 2.2. Physicochemical Characterization

#### 2.2.1. Ash, Humidity, and Carbohydrate Content

Fractions of 2 g of *Sargassum* were placed in previously weighed porcelain crucibles in an oven with natural circulation at 150 °C for 5 h, weighing the crucibles after cooling at room temperature for 30 min in a desiccator with silica gel. The mass obtained after this step was considered the dry bioindicator. The crucibles were then placed in a muffle furnace for 2 h at 600 °C, and the cooling and weighing steps were repeated. The mass obtained after this step was considered to correspond to the ashes of the bioindicator. Measurements were made in triplicate, expressing results as the mean of the three replicates with a confidence interval. The percent of ash content was calculated using the following equation:

$$\mathrm{Ashes}\;(\%)=\frac{w\left(ashes\right)}{w(\mathrm{dry}\;\mathrm{Sargassum})}\cdot 100$$

For determination of the total carbohydrate content (TCC), the biomass was washed several times with distilled water to remove excess salts from the surface. Electrical conductivity was measured with a conductivity meter after each wash until it reached ≤1 mS cm^−1^. Next, 4.0 g of biomass was weighed and placed in 250 mL of distilled water, stirring for 24 h in an orbital shaker. After this first step, a supernatant with polysaccharides (fraction A) was obtained.

Another fraction of 4.0 g of biomass was again weighed, treated with 5% KOH solution (MERCK, analysis quality) and left stirring in an orbital shaker for 24 h at room temperature. The biomass of the supernatant containing polysaccharides was centrifuged and separated to obtain fraction B. After discarding the biomass, fractions A and B were mixed to obtain a single extract for the determination of total carbohydrates. Briefly, 2 mL of this extract was added to 20 mL of distilled water, and 2 mL of this new solution was then mixed with 0.5 mL of 3% aqueous phenol in test tubes, immediately followed by the addition of 5 mL of concentrated H_2_SO_4_ and stirring. The tubes were cooled in an ice bath for 30 min, and the optical density was measured at 490 nm in glass cuvettes using a UV-Vis spectrophotometer with a path length of 1 cm. A standard curve was created by mixing 2 mL of solution containing between 6 and 60 μg of glucose with 0.5 mL of 3% aqueous phenol in test tubes and adding 5 mL of H_2_SO_4_. Controls were prepared with 2 mL of distilled water, 0.5 mL of 3% aqueous phenol, and 5 mL of H_2_SO_4_.

#### 2.2.2. Scanning Electron Microscopy Analysis with X-ray Energy Dispersive Spectroscopy

The sample for this analysis was washed several times with distilled water to remove any salts from the cell wall surface and then dried in an oven with natural circulation at 60 °C. Finally, elements on the surface of samples were qualitatively analyzed using energy-dispersive X-ray scanning electron microscopy (SEM) in a Thermo Fisher Quanta 250 FEG Scanning Electron Microscope (Waltham, MA, USA) equipped with an EDAX-DX-4 energy-dispersive microanalysis system.

#### 2.2.3. Fourier Transform Infrared Spectroscopy (FTIR) Analysis

The sample for this analysis was introduced into a ThermoFisher Scientific Nicolet iS10 FTIR spectrophotometer (Waltham, MA, USA) to obtain the infrared spectrum with ambient background (with corrections for gases present in the atmosphere). Functional groups were determined from the intensities of the bands as a function of the frequency, and band values were compared with the spectra of reference compounds reported in the literature.

#### 2.2.4. Thermal Analysis

Thermal Analysis was performed in a TA Instruments Derivatograph model Q500 using EA Universal Analysis 2000 software (version 5.4). All thermograms were obtained with the simultaneous recording of DTA (differential thermal analysis), TG (thermogravimetry), T (temperature), and DTG (differential thermogravimetry) curves.

TG curves were converted into continuous thermograms using the Windows-compatible software (version 5.4) supplied by the equipment manufacturer, and D1TG thermograms of the first rate of mass change (dm dt^−1^) were also obtained. The reported error for quantitative TG analysis is ±2.00%.

#### 2.2.5. Elemental Content

The Environmental Protection Agency plant tissue digestion procedure was carried out using the solution resulting from the digestion process for simultaneous determination of the elemental content. Measurements were made in an inductively coupled plasma optical emission spectrometer (ICP-OES) and an inductively coupled plasma mass spectrometer (ICP-MS) (CT, USA), both with simultaneous analysis using a solid-state detector. The blank assay was performed under the same preparation conditions as for the digestion procedure. The results were expressed as µg of element per kg of dry material (µg kg^−1^).

#### 2.2.6. Point of Zero Charge (PZC) Determination

PZC is quantified as described by González-Fernández et al., 2021 [21]. The adsorbed proton mass is evaluated by the following equation:

$$q_{H+}=\frac{C_{N}\left(V_{B}-V_{M}\right)}{m}$$

The surface charge (SC) is estimated using the following equation:

$$\mathrm{SC}=\frac{q_{H+}F}{S}$$

The SC of the materials is plotted against pH to obtain the SC distribution. The PZC is the pH at which the SC is neutral (SC = 0).

The term *q_H_*_+_ refers to the amount of moles of proton adsorbed on the adsorbent at a given final pH value (mol g^−1^). *C_N_* is the concentration of the neutralizing solution (mol L^−1^), *V_M_* is the added volume of the neutralizing solution of NaOH or HCl so that the solution with the adsorbent reaches a determined value of final pH (L), and *V_B_* is the added volume of the NaOH or HCl neutralizing solution so that the solution without the adsorbent reaches a given final pH value (L).

#### 2.2.7. Potentiometric Titration: pKa Determination

The pKa of the sorbents was determined using the method proposed by Cuizano and Navarro, 2008 [22] with some modifications. Briefly, 1.0 g of biosorbent pretreated with HCl was added to 50 mL of a KCl solution (0.1 mol L^−1^) to maintain a stable ionic strength throughout the titration. It was titrated with a standardized solution of 0.1 mol L^−1^ of NaOH in the range of pH 2 to around pH 13 using an EasyPlus™ automatic titration instrument from Mettler Toledo (Columbus, OH, USA).

The total concentration of carboxyl groups [*COOH*]*_t_* is calculated with the following equation:

$$\left[COOH\right]t=\frac{Ve\;\left[NaOH\right]}{m}$$

Katchalsky et al., 1954 [23], showed that the titration curve of a polyacid can be described by the following equation, based on the *pK* constant and *n*:

$$pH=\frac{pK\;–\;n\;log\;\left(1-\alpha \right)}{\alpha }$$

where *n* is an empirical constant, whose acceptable value is positive and close to one, and α is the degree of dissociation defined in the following equation:

$$\alpha =\frac{\left[COO^{-}\right]}{\left(C_{0}V_{0}\right)/\left(V_{0}+V_{b}\right)}\;$$

where *V_b_* is the volume of base used and *C*_0_ is the previously calculated initial concentration of acid groups referred to the volume of the solution, with *V*_0_ replacing *m*. The variable [*COO^−^*] can be calculated by using the following equation, where *C_b_* represents the concentration of the titrant:

COO−= H++VbCbV0+Vb−KwH+

*pKa* and *n* values of the adsorbent material were determined by pH versus log (1 − α)/α linear regression analysis.

### 2.3. Cd and Pb Determination in Aqueous Solution

Concentrations of Cd and Pb in aqueous solution were determined by flame atomic absorption spectrometry using hollow cathode lamps and the calibration curve method. A primary standard of 1000 mg L^−1^ of the solutions of each metal was prepared by weighing the corresponding masses of salts of these metals and completely dissolving them in water up to a volume of 1.0 L. A secondary standard was prepared by diluting an aliquot of this solution in deionized water to reach a concentration of 100 mg L^−1^.

Calibration curve standards (Supplementary Materials) of these solutions were prepared in a concentration range of 1.0 to 1000 mg L^−1^. Adjusting the pH accordingly with NaOH and HCl solutions, Cd and Pb concentrations were measured using a VARIAN SPECTRAA 220 Atomic Absorption Spectrometer (Palo Alto, CA, USA).

### 2.4. Experimental Data for the Adsorption Equilibrium of Cd and Pb

Experimental data for the adsorption equilibrium of Cd and Pb were obtained in a batch adsorber using a methodology similar to the one described by González-Fernández et al., 2021 [21].

The mass of Cd or Pb adsorbed on the biomass of *Sargassum* was calculated by using the following formulas:

$$q=\frac{V_{0}C_{0}-V_{f}C_{f}-\sum_{i=1}^{N}V_{i}C_{i}}{m}$$

$$V_{f}=V_{0}-\overset{N}{\underset{i=1}{\sum }}V_{i}+V_{a}$$

where:

*C*_0_ = Initial metal concentration, mg L^−1^
*C_f_* = Final metal concentration, mg L^−1^
*C_i_* = Metal concentration in sample number *i*, mg L^−1^
*m* = Adsorbent mass, g
*N* = Number of samples
*q* = Mass of metal adsorbed per unit mass of adsorbent, mg g^−1^
*V*_0_ = Initial volume, L
*V_f_* = Final volume, L
*V_i_* = Volume of sample number *i*, L
*V_a_* = Total volume of acidic and basic solutions added to adjust the pH of the adsorber solution, L.

The equilibrium of the adsorption process was modeled by fitting the experimental results to the equations of Langmuir, Freundlich, and Radke–Prausnitz (R–P) isotherms. These isotherms can be represented, respectively, by the following expressions:

$$q_{e}=\frac{q_{m}K_{L}C_{e}}{1+K_{L}C_{e}}$$

$$q_{e}=K_{F}C_{e}^{{}^{}1/n}$$

$$q_{e}=\frac{K_{R}C_{e}}{1+a_{R}C_{e}^{\beta }}$$

Data obtained in multicomponent equilibria were evaluated using the non-modified Langmuir multicomponent isotherm (NLMI), extended Langmuir multicomponent isotherm (ELMI), modified Langmuir multicomponent isotherm (MLMI), non-modified Redlich–Peterson multicomponent isotherm (NRPMI), modified Redlich–Peterson multicomponent isotherm (MRPMI) with an interaction factor, extended Freundlich multicomponent isotherm (EFMI), and Sheindorf–Rebuhn–Sheintuch isotherm (SRSI). The equations for these isotherms are exhibited in Table 2.

Parameters of the competitive adsorption models were also obtained by minimizing the MC objective function. The mean percentage deviation (%Des) was calculated as follows:

$$\%Des=100\cdot \frac{1}{k}\overset{k}{\underset{i=1}{\sum }}\frac{q_{ei,exp}-q_{ei,cal}}{q_{ei,exp}}$$

## 3. Results and Discussion

### 3.1. Physicochemical Characterization of Sargassum

Table 3 displays the results obtained for the characterization of *Sargassum* using different analytical techniques. The moisture content of the material was influenced by its nature, as reported by Piña Leyte-Vidal et al., 2019 [20]. The percent moisture was 8.3 ± 0.6, close to the value reported by Silva et al., 2008 [24], for a species of a similar nature. These results allowed the amounts of each bioindicator to be corrected in subsequent experiments according for the actual content of dry bioindicator.

The ash content of the *Sargassum* biomass was also similar to the value observed by Silva et al., 2008 [24], and some association was observed between the ash content obtained by gravimetry and the cationic content determined by ICP-OES and ICP-MS. This ash content corresponds to the amount of inorganic matter in the *Sargassum* biomass.

Results for the total carbohydrate content of the biomass were also comparable to those reported by Kumar et al., 2015 [25], for similar algae species. Carbohydrates have numerous functional groups such as -OH and -COH, among others, which could provide an effective environment for complexation and interaction with cationic metal species and may play a crucial role in their uptake in aqueous solution.

**Figure 1:**
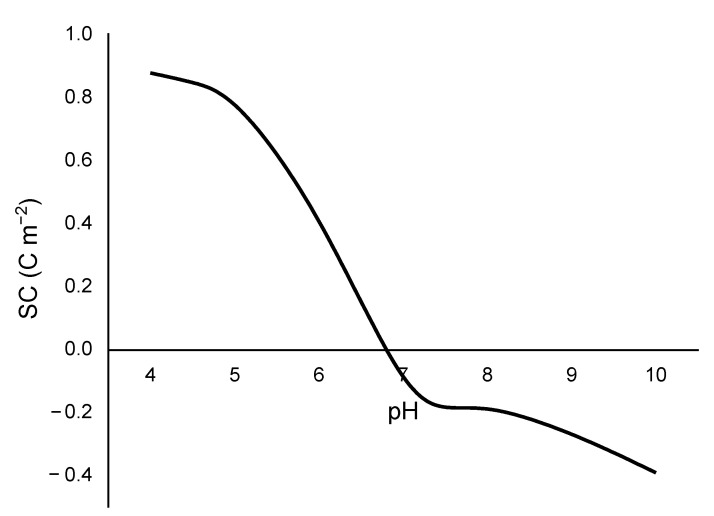
Surface charge curve vs. pH for *Sargassum* biomass.

The PZC obtained for this material was 6.75 (Figure 1), which was very close to the neutral zone of the pH range. Accordingly, this adsorbent is slightly acidic, with a lower concentration of basic than acid sites.

A review of the relevant literature showed that the PZC for this material ranges from 3.6 [26] to 9.0 [27] depending on the species and ecosystem. The present finding is within the range of 6.1 to 7 reported by Ahmady-Asbchin and Jafari, 2012 [28], and Kleinübing et al., 2010 [29]. The PZC value is useful to select pH conditions that favor the electrostatic attraction of metallic species in solution to the adsorbent. Thus, when the pH of the solution is above the PZC, the surface of the material has a net negative charge, promoting its uptake of positive species [30].

**Figure 2:**
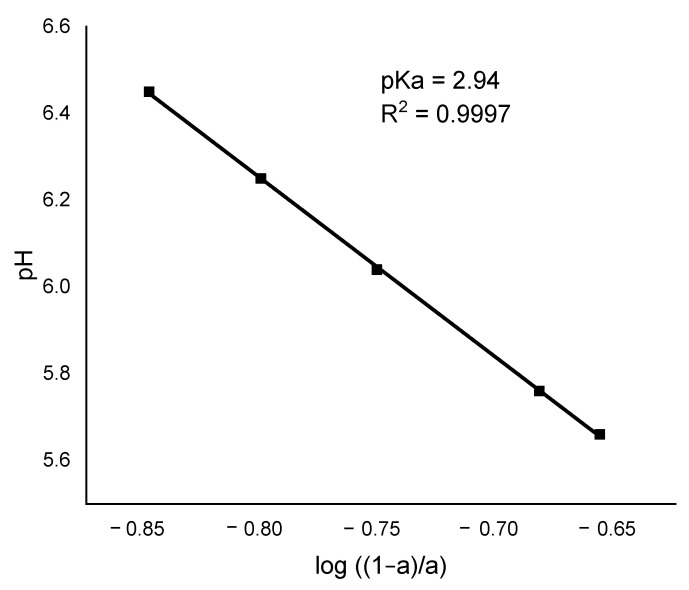
Linear regression to determine the pKa of *Sargassum* according to the Katchalsky model.

The pKa of the material was determined after calculating the parameters needed for the linear regression analysis described by Katchalsky & Miller, 1954 [31]. The result is exhibited in Figure 2.

The pKa was found to be 2.94, which corresponds to the mean pKa of the functional groups in the algal cell wall (e.g., fucoidan and polyalginates) and confirms the high adsorption capacity for heavy metals at pH values > 3 [32,33].

Sheng et al., 2004 [33], observed that the functional groups that bind to heavy metals in marine algae are carboxyl, phosphates, and sulfonic acids in the form of phosphate proteins, carboxylic groups, alginate, and fucoidans, which have pKa values of 3–4, 1.8–2.5, 1–4, and 1–2.5, respectively [32,33]. Unfortunately, it was not possible to evaluate the contribution to adsorption of the hydroxyl group, because it is not ionizable under these experimental conditions.

The concentrations of active sites in the adsorbent are listed in Table 3. The concentration of acid and basic sites agrees with the values reported for *Sargassum* biomass by Tarbaoui et al., 2016 [34], who described five-fold higher concentrations of acidic than basic sites. Their findings for carboxylic, lactonic, and phenolic groups are similar to the present results. The predominance of acidic over basic sites corresponds to the pKa value of the material (weak acid value) and to the PZC of the material, which is slightly acidic.

### 3.2. Elemental Content of Sargassum (ICP-MS and ICP-OES)

**Figure 3:**
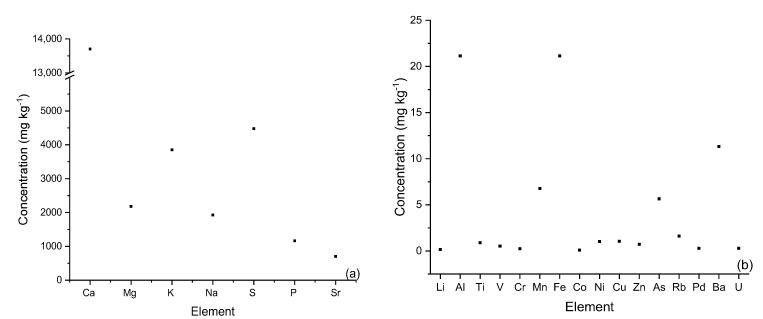
Elemental content (major (**a**) and minor (**b**) elements) of *Sargassum* biomass determined by ICP-MS and ICP-OES.

The concentration of minor elements (a) and major elements (b) is shown in Figure 3. *Sargassum* had high concentrations of sodium, magnesium, and potassium, typical of materials that develop in a saline environment, with cell walls and constituent polymers that are capable of accumulating these elements [24].

There were also high concentrations of sulfur, associated with sulfonic groups and their derivatives in fucoidan, the second most abundant polymer in the algal cell wall [18]. Additionally, there were moderately high concentrations of aluminum and iron that may evidence sustained anthropogenic contamination with sources of these metals.

Other metals such as lithium, cobalt, nickel, copper, and zinc were found at low levels, as also observed by Piña Leyte-Vidal et al., 2019 [20], while the element with the highest concentration in *Sargassum* biomass was magnesium. These findings were consistent with the results of elemental analysis by energy-dispersive spectroscopy (EDS), which showed magnesium to be a major element on the surface of the material.

According to these results, the aforementioned elements almost exclusively determine the cationic content of the material, as reported by Casas-Valdez et al., 2006 [35], Yang and Chen, 2008 [36], and Sierra-Vélez and Álvarez-León, 2009 [37], for the genus *Sargassum*.

### 3.3. FT-IR Spectroscopy

**Figure 4:**
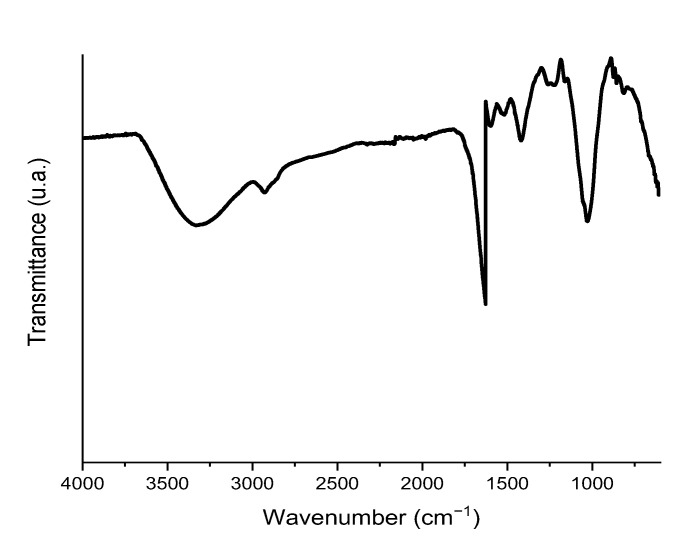
Infrared spectrum of *Sargassum* biomass.

Figure 4 depicts the FT-IR spectra for *Sargassum*, whose large number of bands indicates the highly complex composition of the material, with numerous surface functional groups. Positions of the most representative bands are reported in Table 4 along with their assignments [38], revealing signals associated with the functional groups –COOH, –COO^−^, –OH, and –NH. These functional groups correspond to those most commonly found in the main constituent compounds of the cell wall of brown algae, i.e., alginic acid, alginates, proteins, and polysaccharides [18]. The presence of these functional groups could explain the surface interaction capacity of the adsorbent with the heavy metals in the medium either by electrostatic attraction or by complex formation. Comparable results were described by Piña Leyte-Vidal et al., 2019 [20], for a similar material.

### 3.4. Thermogravimetric Analysis

**Figure 5:**
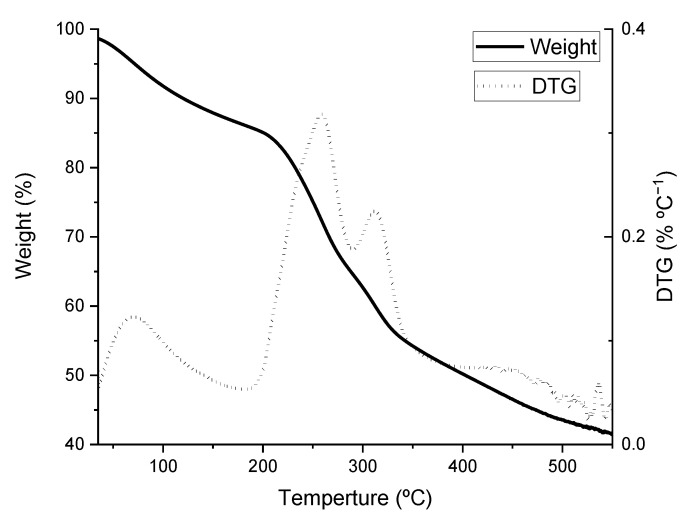
Thermogravimetric curves of *Sargassum* weight loss.

Figure 5 depicts the curves obtained when the sample underwent heat treatments between 35 and 550 °C. The differential curve (DTG) shows a first peak of heat absorption at around 95 °C, corresponding to water evaporation, with no pyrolysis at this stage [39]. The exothermic effect generally appears when the temperature rises above 190 °C, largely due to the breakdown of proteins, soluble polysaccharides, and organic matter [40].

Pyrolysis of carbohydrates (e.g., sugar and starch) begins at 250 °C, with a release of gases such as methane and other derivatives of light hydrocarbons and water vapor due to the presence of sulfated polysaccharides. CO and CO_2_ evolve during this process [41]. Pyrolysis of proteins and amino acids begins above 310 °C, generating gases such as NH_3_ [42].

At more than 350 °C, pyrolysis is still observed as an exothermic reaction and small exothermic peaks are observed. This may be due to the exothermic reaction produced by the participation in pyrolysis of polysaccharides, proteins, and ash residues, among other components. It can also result from the decomposition of residual organic matter and carbonate minerals [43].

### 3.5. Scanning Electron Microscopy

**Figure 6:**
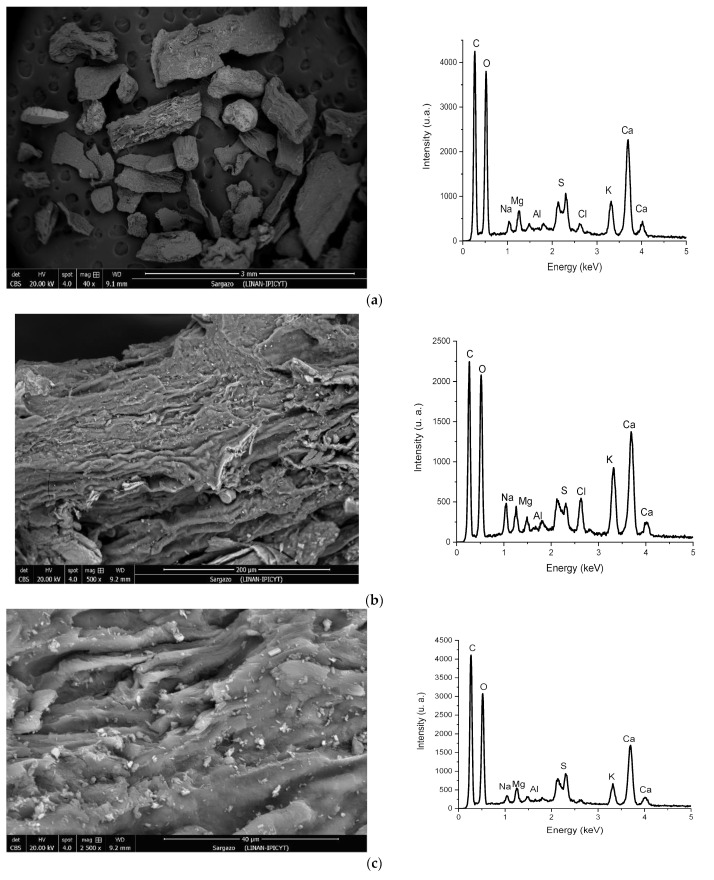
SEM images and EDS spectra of *Sargassum* at: (**a**) 40×, (**b**) 500×, and (**c**) 2500×.

Figure 6 displays the SEM micrographs of the adsorbent at different magnification values and the respective EDS spectra. The micrographs obtained reveal the wide morphological and structural heterogeneity on the surface of the material. Signals corresponding to C, O, Na, S, Mg, Cl, K, Ca, and Al were observed in the EDS spectra. Some of these elements participate in the ion exchange process during biosorption and continue to occupy biomass binding sites, blocking them from other metals [44].

Figure 6 shows that particles of the material have a fractured and rough surface and that their shapes and sizes are highly irregular. The particle size distribution is not uniform, and cavities and channels can be observed. Similar results were reported for species of algal origin by Piña Leyte-Vidal et al., 2019 [20], who described the irregularity and rupture of the adsorbent surfaces, with the appearance of channels.

### 3.6. Monocomponent Adsorption Isotherms

**Figure 7:**
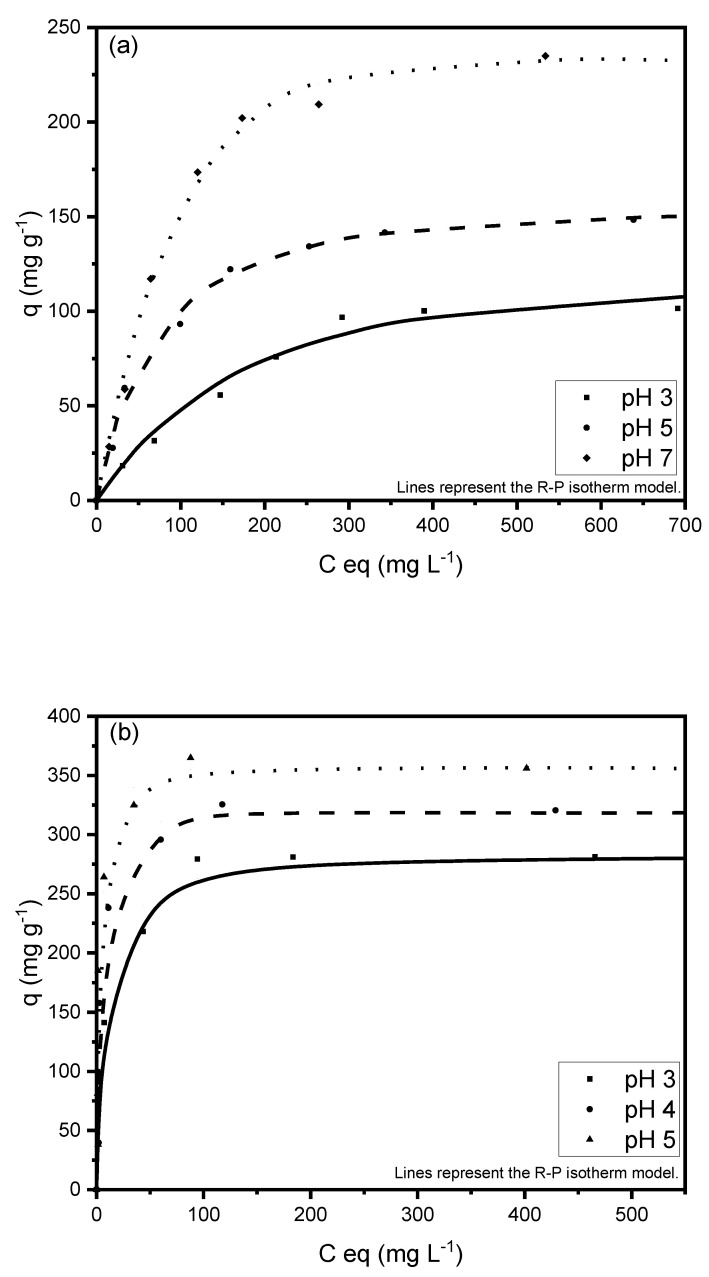
Monocomponent adsorption isotherms for (**a**) Cd(II) and (**b**) Pb(II) at 25 °C.

The adsorption isotherms of Cd(II) and Pb(II) are depicted in Figure 7, showing the experimental data described by the Radke–Prausnitz mathematical model.

This figure reveals two stages. In the first stage, the adsorption capacity increases with a higher metal concentration at equilibrium because the material initially has a large number of active sites for the retention of metal cations. These positions become occupied with increased metal concentration and, at a given concentration, it becomes more difficult for metal cations to be exchanged. In this second stage, a decrease in the slope can be seen, representing saturation, when the maximum experimental adsorption capacity is determined.

**Figure 8:**
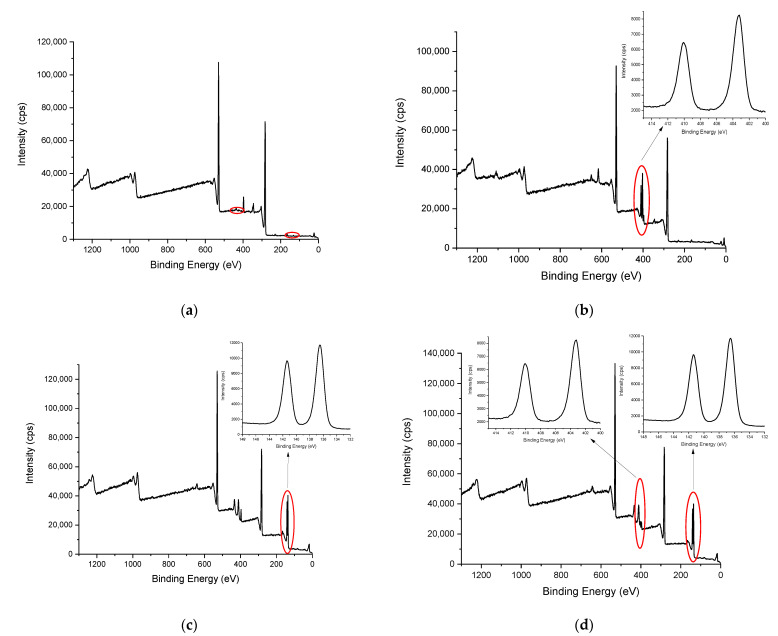
XPS spectra of (**a**) *Sargassum* biomass, (**b**) *Sargassum* biomass after Cd adsorption, (**c**) *Sargassum* biomass after Pb adsorption, and (**d**) *Sargassum* biomass after competitive Cd and Pb adsorption.

Figure 8a shows the XPS spectrum of the natural *Sargassum* biomass and Figure 8b,c show the spectrum of the same material after being used in the adsorption process of Cd and Pb, respectively. In these figures, the presence of these metallic elements in the material after the adsorption process can be corroborated. The same occurs in Figure 8d which shows the XPS spectra of the material used in the competitive adsorption experiments.

In Figure 8a, a peak can be observed in the region close to 349 eV, which corresponds to the 2p calcium line. However, when the material is subjected to the adsorption process (Figure 8b–d) this peak disappears, indicating that calcium is displaced from the biomass and replaced by the adsorbed metals.

Table 5 exhibits the parameters obtained for the isotherm models of monocomponent systems. The quality of the fit was evaluated by the coefficient of determination and the Durbin–Watson statistical test for the correlation of residuals, according to the probability values obtained with 95% confidence (STATGRAPHICS Centurion XV software version 15.12.06).

According to these results, the best fit is obtained with the R–P model for all materials, which showed a β constant very close to 1, indicating that it is adequate for the 3 biosorbent species studied [45]. The value of the R–P constant is also relatively high, indicating a good fit to the model and a favorable adsorption of the analyte on the adsorbent [46].

The adequacy of the theoretical R–P model to describe the experimental isotherms in some metal-adsorbent systems suggests the presence of energetically homogeneous and heterogeneous zones on the surface of the materials for adsorbing metal cations, as established in the precepts of Langmuir and Freundlich models. This behavior was previously reported [47].

The major impact of pH on heavy metal biosorption is well documented [48,49,50], and the pH value is known to influence the protonation of functional groups in a biomass. The results in Figure 7 show that the adsorption capacity for both contaminants increases with higher pH, confirming the strong dependence of biosorption on this value. The cell wall matrix of green algae contains sulfate, carboxyl, and amino groups [42]. At low pH values, repulsive forces between the cell wall ligands and the metal cations produce a decrease in the adsorption capacity. As pH increases, more groups become negative and attract metal ions [51].

The effect of pH can be explained in part by a competition effect between heavy metal and H^+^ ions. At low pH values, H^+^ ions appear in higher concentration in the solution and compete for the binding or active site in the adsorbent. When the pH value is higher, the concentration of H^+^ ions decreases and the metal cations can occupy the active sites easier [52].

### 3.7. Multicomponent Adsorption Isotherms

**Figure 9:**
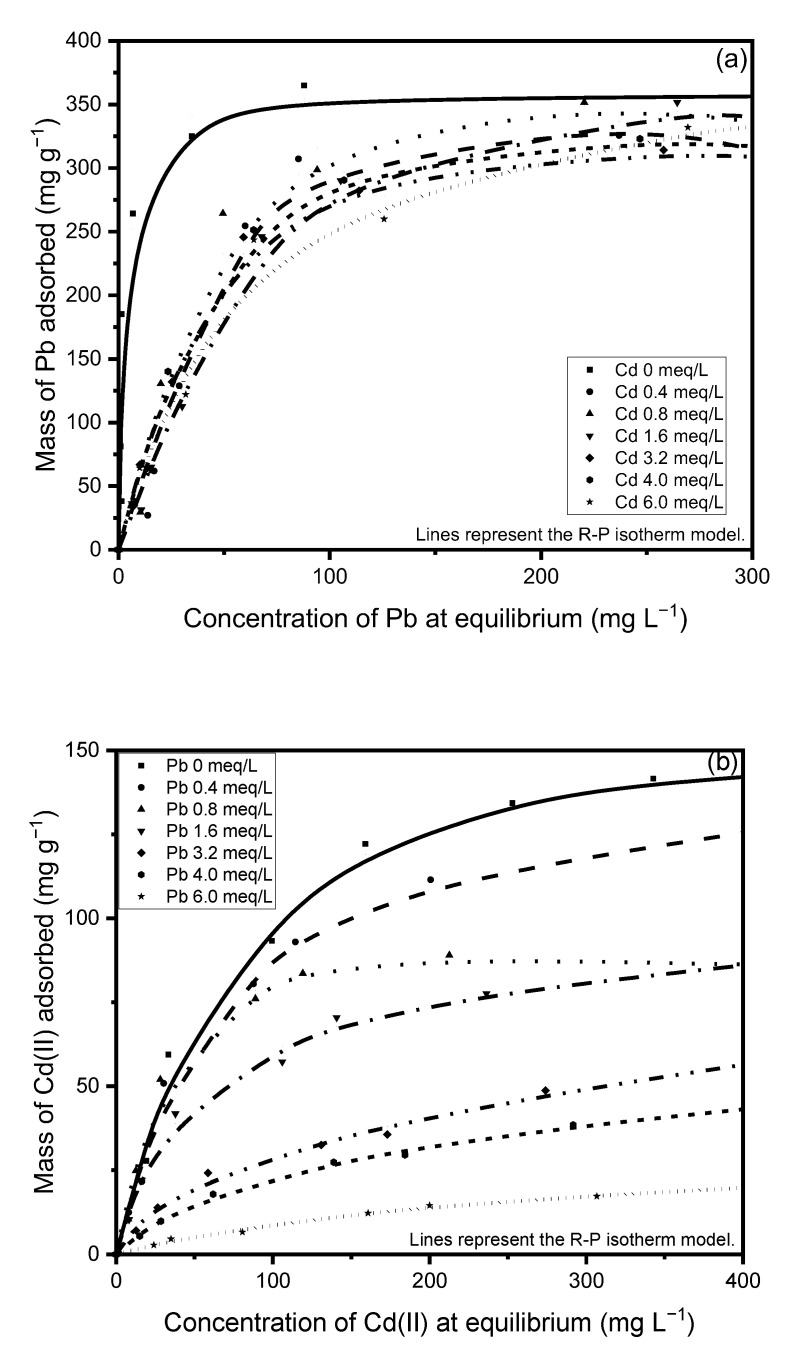
Binary adsorption isotherms of Cd(II)–Pb(II) on *Sargassum* biomass. (**a**) Cd(II) and (**b**) Pb(II) adsorption at pH = 5.0 and T = 25 °C.

Figure 9 depicts the multicomponent isotherms of Cd(II) (a) and Pb(II) (b) in two dimensions. These experimental data are described by the Radke–Prausnitz mathematical model, which yielded the lowest %D values in all cases.

The behavior of the competing isotherms is highly similar to that of the single adsorption isotherm; however, the capacity to remove either Pb(II) or Cd(II) is reduced by the presence of the competitive ion. In the present experiments, the presence of Pb(II) had a major impact on the adsorption of Cd(II), whereas the presence of Cd(II) had a small effect on the adsorption of Pb(II), observing only a slight variation when the concentration of the competitive ion was increased.

**Figure 10:**
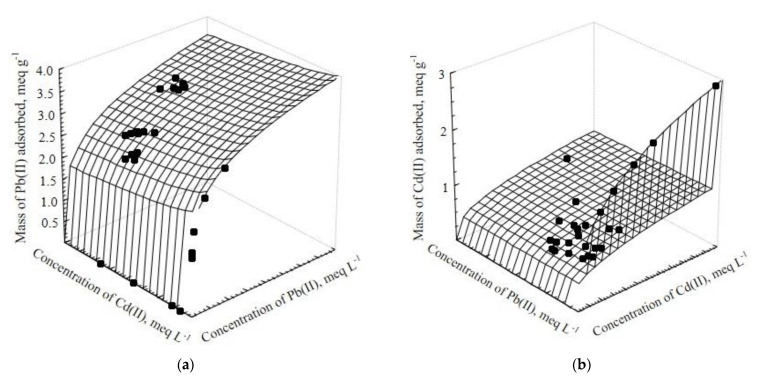
Multicomponent adsorption isotherms of (**a**) Cd(II) and (**b**) Pb(II) on *Sargassum* biomass at T = 25 °C and pH = 5.0. Adsorption surfaces are predicted with the EFMI model.

Figure 10 depicts the experimental data in three dimensions as represented by the EFMI model, which had the lowest %D among the isotherm models fitted to the data.

The dependence of Pb(II) adsorption on the Cd(II) concentration at equilibrium is clearly observed in Figure 10. The adsorption of Pb(II) was vaguely affected by Cd(II). This results show that Pb(II) ions demonstrate higher affinity for the active sites of *Sargassum* biomass than for Cd(II) ions, which is also evidenced by the shape of the single-component isotherms.

The selectivity ratio, S, calculated as reported by Medellin-Castillo et al., 2017 [53], when Pb(II) and Cd(II) concentration are equal to 4.0 meq L^−1^ (adsorption capacities are predicted from the single adsorption isotherms) has a value of 2.42, but it was 5.22 when the adsorption capacities were predicted using the EFMI model. Then, Pb(II) showed strong antagonism in the Cd(II) adsorption, although Cd(II) did not considerably affect the uptake of Pb(II).

This information can be contrasted with that found in the XPS quantification analysis (Table 6). It can be observed that the Cd concentration on the surface is lower than the Pb concentration when the adsorbent is subjected to the competitive adsorption experiments of these two metals.

### 3.8. Effect of Temperature on the Adsorption of Cd(II) and Pb(II) by Sargassum

**Figure 11:**
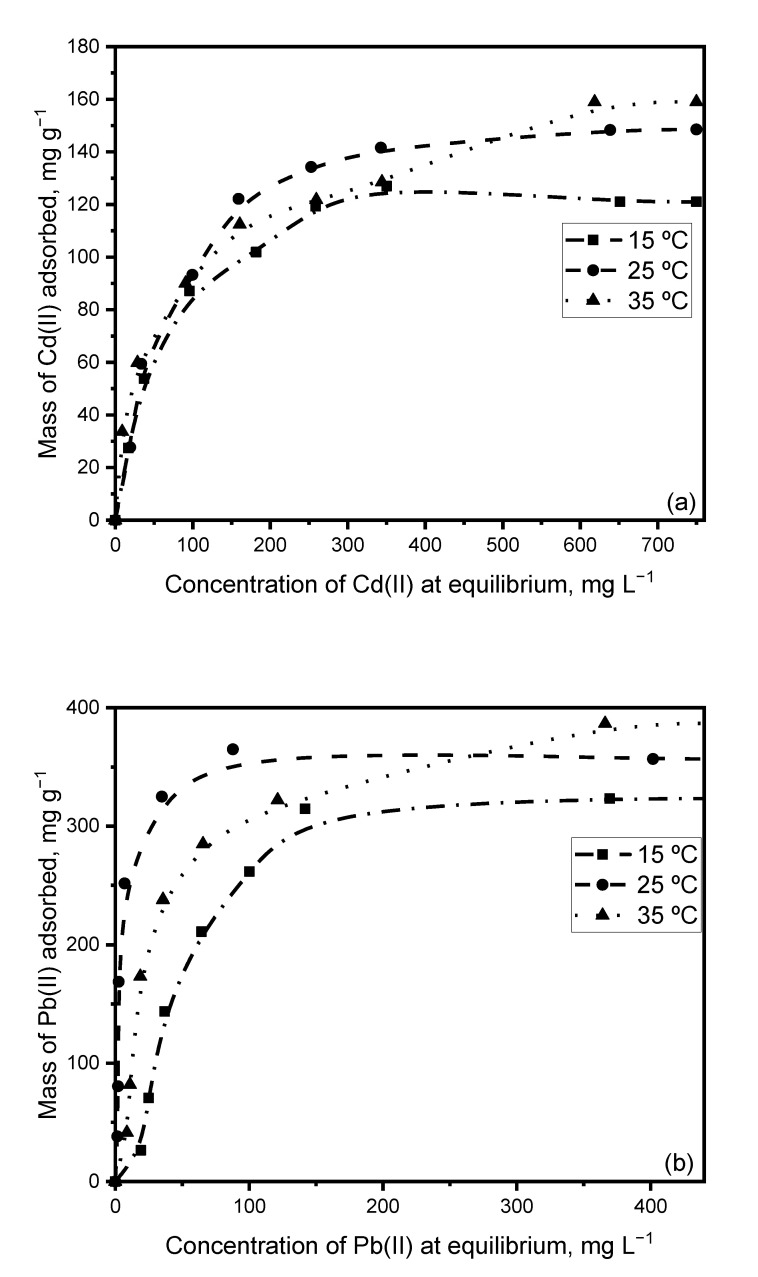
Effect of temperature on the adsorption of Cd(II) (**a**) and Pb(II) (**b**) on *Sargassum* biomass.

Figure 11 shows the effect of temperature on the adsorption capacity of Pb(II) and Cd(II) on *Sargassum* biomass. The monocomponent adsorption procedure was followed under optimized conditions at 15 °C and 35 °C.

Figure 11 shows that a rise in temperature during the sorption process increases the adsorbent capacity throughout the studied range. In the present case, the enthalpy variation is positive, being an endothermic adsorption process. Similar results were obtained by Aksu, 2001 [51], for Cd adsorption on *C. vulgaris* and by Aravindhan et al., 2009 [54], for dye removal on *Caulerpa scalpelliformis*.

Determination of the entropy or enthalpy of the sorption process is key to establish whether or not the sorption process is spontaneous from a thermodynamic standpoint. The adsorption of Pb(II) on biomass, as in the case of Cd(II), can be assumed to be in reversible heterogeneous equilibrium expressed by:

$$Pb^{2+}\left(aq\right)\overset{\;}{↔}\;Pb^{2+}-algae\;biomass$$

Gibbs free energy (Δ*G***°**) for this equilibrium can be determined by the following equation:

$$\Delta Gº=-RTlnK_{c}^{0}$$

where *R* is the universal constant for gasses (8.314 J mol^−1^ K^−1^), T is the absolute temperature (*K*), and $K_{c}^{0}$ is the equilibrium constant.

The relationship between $K_{c}^{0}$ and temperature is presented in the Van ’t Hoff equation:

$$lnK_{c}^{0}=\frac{\Delta Sº}{R}-\frac{\Delta Hº}{RT}$$

The sorption entropy change, Δ*S***°**, and the enthalpy change, Δ*H*º, can be obtained from the slope and intercept of the regression line of ln$K_{c}^{0}$ versus 1/*T*. These parameters were calculated by using the Langmuir isotherm, replacing the equilibrium constant $K_{c}^{0}$ with the Langmuir constant (*K_L_*).

Table 7 shows the thermodynamic parameters of Pb(II) and Cd(II) adsorption at equilibrium at the three selected temperatures.

According to the thermodynamic treatment of Pb(II) and Cd(II) adsorption on the biomass, the ∆*G*° value was negative at all three temperatures, indicating that chemisorption is a favorable and spontaneous mechanism underlying sorption of the ions on biomass [47]. The positive ∆*H*° value reflects the endothermic nature of the process and verifies that this adsorption process is largely governed by chemisorption [55].

The relatively high modular ∆*H*° values reaffirm the possibility of a chemisorption process [56,57]. For their part, the positive ∆*S*° values evidence the randomness of the solid–solution interface during Pb(II) and Cd(II) adsorption [58] and indicate the affinity of the adsorbent for ions in solution [59,60].

## 4. Conclusions

Physicochemical characterization of the biomass of the genus *Sargassum* collected from Mexican Caribbean shores revealed that both the morphology and the chemical composition of the cell wall favor its incorporation of metallic element ions.

A study of the equilibrium and thermodynamic treatment of adsorption showed the presence of adsorption processes during the retention of Pb(II) and Cd(II) on the natural biomass of *Sargassum*.

The Cd(II) and Pb(II) adsorption capacities are slightly above 240 mg g^−1^ and 350 mg g^−1^, respectively, which are higher than values previously reported for materials of a similar nature and for some conventional and novel adsorbents.

When both chemical elements are simultaneously present in solution, the *Sargassum* biomass displays a higher affinity for Pb(II) than Cd(II) cations. Pb(II) therefore shows strong antagonism in the competitive adsorption of Cd(II), whereas Cd(II) has no significant effect on the competitive adsorption of Pb(II).

Thermodynamic treatment of Pb and Cd adsorption on the biomass revealed negative ∆*G*° values at all temperatures, indicating a spontaneous process, while the positive ∆*H*° values affirm its endothermic nature and the positive ∆*S*° values evidence the affinity of the adsorbent for Pb(II) and Cd(II) ions in solution.

The present findings demonstrate that *Sargassum* biomass is an excellent option for the removal of heavy metal elements such as Pb and Cd in comparison with more traditional or even novel adsorbents. The composition and physical and chemical properties of this biomass make it a highly promising adsorbent whose utilization could contribute to reducing the damage it currently causes to the economy and coastal ecosystems of the Caribbean region of Mexico.

## Figures and Tables

**Table 1:** Adsorption capacities for Pb(II) and Cd(II) from different conventional and novel adsorbents.

Adsorbent	Adsorbate	Adsorption Capacity (mg g^−1^)	Refs.
Carbon aerogel	Pb(II)	34.72	[5]
Carbon nanotubes		12.41	[6]
Bentonite		15.38	[7]
Bituminous coal		8.89	[8]
Red mud		64.79	[9]
Olive branches activated carbon (AC)	Cd(II)	38.17	[10]
Commercial AC		25.13	[11]
Nanocomposite mesoporous silica		148.32	[12]
Red algae *Galaxaura oblongata*		85.5	[13]
Green algae *Ulvalactuca*		29.2	[14]

**Table 2:** Equations for the isotherm models used to describe experimental data in multicomponent systems.

NLMI	$q_{i}=\frac{q_{m}K_{i}C_{i}}{1+\sum_{j=1}^{N}K_{j}C_{j}}$	ELMI	$q_{i}=\frac{q_{max}K_{E,i}C_{i}}{1+\sum_{j=1}^{N}K_{Ej}C_{j}}$
MLMI	$q_{i}=\frac{q_{m,i}K_{i}\left(C_{i}/\eta_{i}\right)}{1+\sum_{j=1}^{N}K_{j}(C_{j}/\eta_{j})}$	NRPMI	$q_{i}=\frac{a_{i}C_{i}}{1+\sum_{j=1}^{N}b_{j}C_{i}^{\beta_{i}}}$
MRPMI	$q_{i}=\frac{a_{i}\left(C_{i}/\eta_{i}\right)}{1+\sum_{j=1}^{N}b_{j}{\left(C_{j}/\eta_{j}\right)}^{\beta_{j}}}$	SRSI	$q_{i}=k_{i}C_{i}{\left(\overset{N}{\underset{j=1}{\sum }}a_{ij}C_{j}\right)}^{\left(\frac{1}{n_{i}}\right)-1}$
EFMI	$q_{1}=\frac{k_{1}C_{1}^{\left(\frac{1}{n_{1}}\right)+x_{1}}}{C_{1}^{x_{1}}+y_{1}C_{2}^{z_{1}}};\;q_{2}=\frac{k_{2}C_{2}^{\left(\frac{1}{n_{2}}\right)+x_{2}}}{C_{2}^{x_{2}}+y_{2}C_{1}^{z_{2}}}$		

**Table 3:** Physical and chemical characteristics of *Sargassum*.

TCC (ppm)	PZC	pKa	Moisture (%)	Ash (%)	Basic Sites (meq g^−1^)	Acid Sites (meq g^−1^)			
						Totals	Carb.	Lact.	Fen.
544.5	6.75	2.94	8.3 ± 0.6	22.2 ± 0.4	0.0836	1.3531	0.7355	0.1267	0.4909

**Table 4:** Main assignments of the FT-IR spectrum of *Sargassum*.

*Sargassum*	
ῡ/cm^−1^	Assignment
3350	v^asoc^_OH_ + v_N-H_
2900	v_Csp3-H_
1650	v^as^_COO_^−^
1400	v^s^_COO_^−^
1050	v_CO (-OH)_

**Table 5:** Parameters of adsorption isotherm models.

Model	Parameter	Cd			Pb		
		pH 3	pH 5	pH 7	pH 3	pH 4	pH 5
Langmuir	*q_m_* (mg g^−1^)	140.6	169.7	287.8	286.7	332.5	366.9
	*k_L_* (L mg^−1^)	0.005	0.013	0.011	0.129	0.179	0.304
	R^2^ (%)	95.5	98.6	97.7	97.9	93.4	89.1
Freundlich	*K_F_* (mg g^−1^)(L mg^−1^)^1/n^	6.3	19.5	21.6	84.1	109.6	139.8
	n	2.2	3.0	2.5	4.5	5.0	5.5
	R^2^ (%)	87.1	88.7	87.2	85.7	75.3	70.5
Radke–Prausnitz	$K_{R}$ (L g^−1^)	0.64	2.06	2.27	36.19	54.66	105.9
	a_R_	0.001	0.008	0.001	0.135	0.140	0.267
	β	1.17	1.07	1.27	0.99	1.03	1.01
	R^2^ (%)	97.0	98.9	99.2	98.0	93.7	89.2

**Table 6:** Quantification of elements on the surface of *Sargassum* biomass used in competitive adsorption experiments.

Peak	Position (eV)	Atomic Concentration (%)	Mass Concentration (%)
C 1s	283	66.07	48.58
O 1s	530	29.75	29.14
N 1s	397	1.76	1.51
Ca 2p	346	0.29	0.71
S 2p	167	0.60	1.18
Pb 4f	136	1.44	18.22
Cd 3d	403	0.10	0.66

**Table 7:** Thermodynamic parameters of Pb(II) and Cd(II) adsorption on *Sargassum* biomass.

T (°C)	Δ*G*° (kJ/mol)		∆*H*° (kJ mol^−1^)		Δ*S*° (kJ mol^−1^ K^−1^)	
	Cd	Pb	Cd	Pb	Cd	Pb
15	−1.54	−3.32	24.35	37.06	0.09	0.14
25	−2.31	−4.44				
35	−3.34	−6.12				

## Data Availability

Data available on request due to restrictions (privacy).

## Supplementary Materials

The following supporting information can be downloaded at: https://www.mdpi.com/article/10.3390/ijerph20032559/s1. Figure S1: Calibration curve standards of Cd and Pb in aqueous solutions.
