# Supplementary material for: Valorization of Sargassum Biomass as Potential Material for the Remediation of Heavy-Metals-Contaminated Waters
Source: Int J Environ Res Public Health. 2023 Jan 31;20(3):2559. doi: 10.3390/ijerph20032559 (PMC9915137; doi:10.3390/ijerph20032559)
Supplement: Supplementary file 1 [file ijerph-20-02559-s001.zip › ijerph-2141373-supplementary.pdf]

SUPPORTING INFORMATION

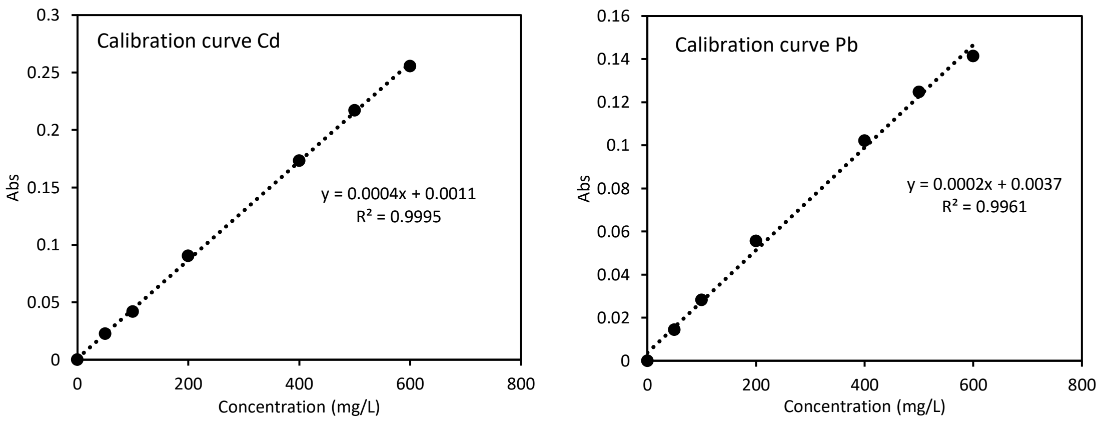

Figure S1. Calibration curve standards of Cd and Pb in aqueous solutions
